# Aluminum exposure from food in the population of Lebanon

**DOI:** 10.1016/j.toxrep.2020.08.018

**Published:** 2020-08-22

**Authors:** Sarine EL Daouk, Alain Pineau, Mariam Taha, Raed Ezzeddine, Akram Hijazi, Mohamad Al Iskandarani

**Affiliations:** a Targets and Drugs of Immunity and Cancer Infections (ERATU - EA 1155 IiCiMED IFR 26), Nantes University, France; b Platform for Research and Analysis in Environmental Sciences (PRASE), Doctorate School for Science and Technology, Lebanese University, Lebanon; c Food and Water Analysis Department, Faculty of Public Health, Lebanese University, Lebanon

**Keywords:** Aluminum, Daily dietary exposure, E-FFQ, FAASs, Food matrix, Provisional Tolerable Weekly Intake

## Abstract

Widely used for a variety of applications, levels of dietary aluminum (Al) have seen a perpetual rise in Lebanon, leading to noticeable effects upon the human body. This study aims to estimate the rates of Al contaminated food consumption and quantify the Al present in different dietary matrices, revealing the major contributors to Al exposure for the Lebanese population. A cross-sectional study was conducted using a customized, self-reported Electronic Food Frequency Questionnaire (E-FFQ) using Curve®, targeting individuals between the ages of 18 and 64 from different Lebanese regions, distributed proportionally. The selection of food was based upon the results of the French EAT2 study. Al levels in food were analyzed using Flame Atomic Absorption Spectrometry (FAAS) after acid digestion. The E-FFQ was completed by 167 respondents. Data analysis was performed on SPSS version 25. Additionally, 97 food items were studied in 2018. Al levels had a mean of 3.56 ± 2.08 mg/kg (ranging from 0.14 to 9.37). The highest Al levels were found in vegetables, followed by sauces and condiments, candies, and ready meals. The Provisional Tolerable Weekly Intake (PTWI) of Al was set at 0.50 mg/kg body weight (60 Kg/person). Al mean Daily Dietary Exposure (DDE) was estimated to be 4341.18 μg/day, with the highest food exposure coming from lettuce, soft drinks, ice cream and tea. Al ingestion rates for the adult Lebanese population does not exceed the international established thresholds of tolerable intake (1 mg/kg/week). National recommendation should be developed to control the presence of metal for food safety purposes.

## Highlights

• Estimation of dietary aluminum rates in the Lebanese population.
• Creation of food frequency questionnaire targeting aluminum.
• Safety requirements and recommendations.

## 1 Introduction

Aluminum (Al) is known to be the third most abundant constituent element of the Earth's crust. Naturally, acid rain can increase Al concentration in soils to toxic levels. Consequently, it can then be found in plants, food and underground water sources, including drinking water [[1], [2], [3], [4]]. In business markets, the metal is widely used in various applications; the production of primary aluminum is increasing worldwide, rising by 40 % between 2008 and 2018 [5,6], reflecting responses to the ever increasing consumption of Al. Industrial food packaging (beverage boxes, baking trays, etc.) accounted for up to 20 % of Al use [7]. As a result, industrial products and processed foods were considered as the first source of food contamination by the metal [[8], [9], [10]].

Al contamination occurs even within the normal preparation of our food. Dyes, anti-caking agents and firming agents are all authorized food additives based on Al that constitute part of the food industry [11,12]. Some, like Al silicate, are used as anti-caking agents. Al sulfate is used as a firming agent and has no Codex provision, whereas maximum levels have been set for the use of Al ammonium sulfate in food production in its various functions as an acidity regulator, color retention agent, firming agent, raising agent and stabilizer [13]. From a legislative point of view, no maximal dose of quantification for Al in food has been determined. Therefore, based on toxicological studies and exposure, in 1990 the Joint FAO/WHO Expert Committee on Food Additives (JECFA) set a Provisional Tolerable Weekly Intake (PTWI) of 7 mg/kg body weight (bw). This was later withdrawn in 2008 to be replaced by 1 mg/kg bw from all sources of Al [14,15]. In 2011, the Committee made a further revision, adding a recommended PTWI of 2 mg/kg bw for all sources of Al for food additives containing Al to the Codex General Standards of Food Additives [15,16]. More recent reports from the Committee recommended the adoption of new Al containing food additives provisions in line with the revision of exceeded PTWI from various studies [17]. The accumulation of Al in the body is associated with oral, inhaled, cutaneous and intramuscular exposures to substances rich in Al [[18], [19], [20]]. This exposure can become toxic in higher doses [21,22]. However, the 67th JECFA (2006) also concluded that Al compounds have the potential to affect the reproductive system and developing nervous system at doses lower than those used in establishing the previous PTWI [15].

Clearly defined Al levels in food could help establish preventive measures regarding the proper use of Al in food preparation, define a national recommendation of Al levels for the general public, and set food Al level control management and regulation measures for the market [23]. Few studies have been carried-out concerning the presence and the detection of metals in food, and the Lebanese population’s exposure to such metals [[24], [25], [26], [27]]. Until now, and to the best of our knowledge, only one study has tackled Al, its presence in soil and the effect on Fattoush salad components [28]. However, in Lebanon, Al use is well documented; it is found in cooking and kitchen utensils for home use, in restaurants and other food related businesses (baking sheets, trays, pans, pots, coffee percolator and others). Furthermore, food comes into contact with Al through packaging (cans, foil, containers, lids, capsules, tubes, composite material for sachet and others) and industrial processes. Some traditional Lebanese foods are prepared, cooked and heated several times using Al utensils (Kanafeh, Maamoul, Foul, homemade tomato sauce, Falafel, Shawarma and many others). We have integrated these foods known to be in contact with Al in our study in order to disclose any consequences for ingestion. In order to have a homogeneous and comparative analysis, we have based our food selection on the results of the second French Total Diet Study (EAT2), which summarized the major presence of Al in vegetables, compotes, infant milk and cereal products [29]. In 2012, the European Food Safety Authority (EFSA) discontinued the use of Al additives in many methods of food preparation due exceeding established PTWI and exposure to Al by certain groups within the population, including children in particular. The EFSA also highlighted that dairy products, particularly cheeses, cereals, starch-based desserts, bread-type products, confectioneries, mixes for bread and bakery wares, and others, are the main contributors of dietary Al.

Food control systems in Lebanon are governed by nine agencies with overlapping functions and a lack of accountability. Food safety practices do not conform to international standards, nor do they ensure the safety of Lebanese consumers [30]. Due to the lack of a proper food control system, no previous recommendation on the use of Al in food was set. Our study aims to quantify Al in different dietary matrices, expose the most consumed food with high levels of Al, and to estimate the average daily intake of Al.

## 2 Material and methods

### 2.1 Study population and assessment of Al-based food consumption

To assess the consumption of foods within the Lebanese market, we have considered the results of the French EAT2 study, implemented between 2006 and 2010 [29]. The selection of food items was based on items previously identified as containing Al, available in the Lebanese market, and traditionally and actually consumed by the Lebanese population. Based on these considerations, a list of 67 food items was compiled as part of an Electronic Food Frequency Questionnaire (E-FFQ) to analyze the consumption of selected food items by the Lebanese population, with the portion sizes of each item included in the E-FFQ following the recommendations of the Lebanon Food Based Dietary Guideline (FBDG) adopted guidelines by the Lebanese Ministry of Public Health (MoPH) and the Food and Agriculture Organization of the United States (FAO) [31] – Table 2. The semi-quantitative E-FFQ entailed socio-economic characteristics and questions related to selected food that comprise frequency of selected items (on daily, weekly and monthly bases). In order to calculate food consumption, the conversion of frequency followed the study model of the European Prospective Investigation into Cancer & Nutrition (EPIC). Therefore, a frequency factor was used as follows: 0 to less than once a month; 0.07–1 to 3 times a month; 0.29–1 to 3 times a week; 0.71–4 to 6 times a week; 1 to once a day [32]. Portion size and food items were grouped in assembled photos to ease recognition of items and minimize biases [33]. The E-FFQ was pilot tested before being used, targeting various population groups aged between the ages of 18 and 64. A customized web-based platform registered under Curve® was created, tested and used to ease data collection and analysis. This platform was used to deliver the E-FFQ, hosted the participants’ answers, and allowed the rapid conversion of coded questions to analytical software, minimizing coding errors [34]. Dissemination of the survey link was done prior to food sampling by a media agency through a messenger service message sent to smartphones. Messages were proportionally segmented by age to cover the official provinces (Beirut, Mount Lebanon, South (including Nabatiyeh), North (including Akkar) and Bekaa (including Hermel)) [35]. The collected information was exported after one week to an excel spreadsheet and thereafter was analyzed using IBM SPSS software version 25.

### 2.2 Food sampling and preparation

Food samples were randomly collected from Beirut market. We purchased 105 packed (N = 70) and unpacked (N = 35) food items between May and September of 2018. Selected items were not limited by seasonable factors. The number of samples in different food groups followed the weighting of Lebanese food consumption patterns study conducted in 2004 [36]. To avoid any cross-contamination with other metals and trace elements from food containers, plastic bags and plastic cutlery were used in all specimen sampling and food handling. Preparation of specimens was performed at the Lebanese University Platform for Research and Analysis in Environmental Sciences at the Doctoral School of Science and Technology (PRASE). Specimens were divided into liquid and solid matrices. A fresh weight of 5 g was retrieved from solid matrices after being mid cut longitudinally, homogenized and placed into porcelain crucibles. Liquid matrices were directly weighted in porcelain crucibles. Dry-ash method, using a digital WiseTherm muffle furnace (Merk, Daihan), was performed; for solid matrices, the temperature was set to 550 °C for 3−4 hours while, for liquid matrices, the temperature was gradually increased to 120 °C, 200 °C, 350 °C, 500 °C, 550 °C at 0′, 60′, 120′, 180′, 210′). For the determination of Al levels, samples were transferred into a 50 mL polytetrafluoroethylene (PTFE) beaker and the following digestion procedure was performed; 1:1 (v:v) H_2_O_2_ (30 %): HNO_3_ (65 %) was added slowly in total portions of 15 mL. The samples were then heated on a hot plate until the solution became clear (15–30 mn). After the solution was cooled and sonicated, it was filtered using 0.45 μm pore size into a lidded conic graduated plastic tube and diluted with deionized water to the mark of 20 mL. Samples were prepared and stored at 4 °C in a darkroom until analyzed progressively in batches. Analyses were performed in an analytical laboratory of the Industrial Research Institute (IRI) accredited by National Accreditation Board (ANAB – ANSI). The American Organization of Analytical Chemist International (AOAC) standard protocols for specimen preparation and analysis were followed.

### 2.3 Metal analysis

#### 2.3.1 Instruments

The analysis of Al was performed using Flame Atomic Absorption Spectrometry (FAAS) (Shimadzu AA-6800 equipped with ACS 6100 auto sampler, Japan) by means of a 309.3 nm Al lamp wavelength and 0.7 nm Slit width. Nitrous oxide (N_2_O) was used as oxidant gas. The operating parameters for the working element were set as recommended by the manufacturer as shown in Table 1.

**Table 1:** FAAS operating parameters for the determination of Al, Shimadzu cookbook.

Instrument settings and analytical conditions of FAAS for the determination of Al				
Step no.	Temperature ^o^C	Ramp time, seconds	Heat	Internal N_2_ flow L/min
1	60	3	RAMP	0.10
2	120	20	RAMP	0.10
3	250	10	RAMP	0.10
4	900	10	RAMP	1.00
5	900	10	STEP	1.00
6	900	3	STEP	0.00
7	2600	3	STEP	0.00
8	2600	2	STEP	1.00

#### 2.3.2 Reagents and glassware

Ultra-pure water, obtained from an Evoqua LaboStar 2 purification device (Semens, UK) was used throughout the experiment. All of the chemicals used for digestion procedures (HNO_3_ and H_2_O_2_) were purchased from VWR (BDH, France). The benchtop was pre cleaned with H_2_O_2_ (30 %). Laboratory glassware and plasticware (beakers, crucibles, tips, tubes and others) were kept overnight in 20 % (v/v) HNO3 solution and then washed, first with distilled water and finally with ultra-pure water before use to prevent any source of contamination in the laboratory. Interference check solution of the multi-component standards, including Al, was purchased from High Purity Standards Greyhound USA at a concentration of 500 μl/mL in 2% HNO3 Tr HF. The latter was used as a stock solution for setting the calibration curve. Control samples were analyzed together with the test samples in an analytical run to control and evaluate the analytical method. The equipment used for the method (such as the balance, oven and glassware) were regularly calibrated and monitored.

#### 2.3.3 Validation

Validation experiments were carried out in order to assess the performance of the method through the determination of recovery, precision, accuracy, linearity, limit of detection (LOD), limit of quantification (LOQ) and uncertainty. Recoveries of Al were determined by spiking known amounts of Al standards of five concentrations 2, 5, 10, 15, 20 ppm. The recovery percentage ranged between 87.52 % and 88.1 %. The quantification of the results was done by using an external standard calibration curve, ranging from 0.1–10 ppm constructed before analysis of the samples, and linear regression equations were used to quantify Al in selected samples. The calibration curve showed good linearity with the coefficient of determination r^2^> 0.9942. LOD was 0.04 ppm and LOQ was 0.12 ppm were estimated from the standard deviation of the twelve blank measurements (LOD = 3 x SD and LOQ = 10 x LOD). The accuracy was performed from the mean value of Certified Reference Material (CRM) containing a certified amount of Al 0.4 ppm. Our obtained results show that our method has high accuracy (over 97 %). The precision of our analytical method was determined from standard deviation of reproducibility using CRM. Our results showed a satisfactory precision 95.5 % for tested samples. In terms of uncertainty, we observed that expanded measurement of uncertainty was 2.00 ± 0.05 ppm, estimated by combining precision with bias of CRM.

#### 2.3.4 Estimate of Al daily dietary exposure and contribution to the PTWI

For the estimation of daily dietary exposure (DDE) of Al for each food item, we have multiplied the concentration of Al in selected food items (mg/kg) with their mean consumption per day retrieved from the results of E-FFQ with the weight of each portion size (g). The sum of all items ended with the below DDE using the equation:

Daily dietary exposure (mg/day) = Σ [element food content (mg/kg) × food intake (g/day)] /1000

To estimate the dietary exposure by bw, we have considered a default body weight of 60 kg for both females and males [37], knowing that no standard weight had been fixed for the Lebanese population [38], but to our knowledge previously reported studies had reflected similarities to American and Western European standards. Additionally, the chosen default weight meets comparative purposes with other international studies [29]. The mean consumption of Al in food was estimated by its 50th percentile and, for higher consumption rates, by its 95th percentile. To calculate the PTWI, we have followed the WHO and EFSA dietary exposure assessment principle [26]. The same formula of DDE was used multiplying by 7 days and counting the body weight.

#### 2.3.5 Food groups

Selected food items were in direct contact with Al in different forms. Some of them were prepared using Al utensils (oven tray and cooking pot) [39], while others are known to contain Al-based food additives or used packages that include Al. They were known to be part of the traditional Lebanese market and/or available in the Lebanese market. Packed and unpacked food were listed under different food groups to create a total of 18 groups. The major analyzed food items for each group were listed as follows: Arabic Sweets (kanafeh; maamoul mad with pistachios, dates and walnuts - all baked in Al trays); Bread and Pastry (Arabic bread, baguette, pizza, fatayer, thyme (zaatar) mankoushe); Cake, Croissant and Biscuits (chocolate muffin, croissant, chocolate wafer); Candies (packaged); Charcuterie (mortadella chicken, turkey and beef and hotdogs); Chicken, Fish and Red meat (rotisserie and grilled chicken, fried escalope, grilled and canned sardines, barbecue); Coffee, Cocoa and Tea (capsuled coffee, Turkish coffee, sachet instant coffee, hot chocolate, cocoa powder and tea); Dairy Products (full fat liquid milk, powdered milk, labneh, yogurt (ready to drink), unsalted packed butter); Dessert, Cream and Jam (packed whipped cream, sweetened condensed milk, cooking cream, packed ice cream, canned strawberry jam); Fruits (fresh apple, plum and canned fruit salad); Juice (pineapple juice, orange juice - packed and canned, and powdered juice); Legumes (cooked foul (fava beans), cooked hummus (chickpeas), canned lentils, canned corn); Potatoes (packet of chips with different flavors, fried, grilled and baked potatoes), Processed Cheese (melted and processed); Ready Meals (shawarma, falafel, basterma, makanek (beef sausages), cooked rice from market); Sauce and Condiments (ketchup, cucumber pickles, onion sauce for rotisserie chicken, hot chili sauce, tomato paste, cinnamon spices, chicken broth cubes); Soft Drinks (canned orange, cola and lemon); vegetables (fresh carrots, lettuce, tomatoes, canned peas and canned mushroom).

#### 2.3.6 Statistical analysis

The statistical analysis has been conducted on SPSS version 25 after importing and cleaning data. Missing values were less than 10 %. Variables were analyzed using ANOVA, one sample *t*-test and independent *t*-test. They were presented in the form of mean and standard deviation. P-value of less than 0.05 was considered significant.

## 3 Results

Out of 167 participants, 37.1 % were males and 62.9 % were females. More than half of the participants were aged below 30 years old (55 %). For the distribution of participants by provinces, our participants came from different areas with the majority (around 45 %) coming from Beirut. For the association with mean Dietary Weekly Intake (DWI), there was no significant difference between genders, age groups or regions. The mean DWI of 0.5 Al (mg/kg bw/day) was statistically different from the EFSA reference value, p < 0.001 –Table 2.

**Table 2:** Characteristics of the E-FFQ participants and their Dietary Weekly Intake (DWI) to Al.

Characteristics of the E-FFQ participants		Dietary Weekly Intake (DWI) of Al (mg/kg bw/day)			
	N (%)	Mean ± SD*	CI 95 %*	Min-Max	P value
Reference value of EFSA* (1 mg/kg bw /week)		0.50 ± 0.27	0.46−0.54	0.11−1.66	0.001
**Gender**					0.558
Male	62 (37.1 %)	0.49 ± 0.25	0.42−0.55	0.12−1.15	
Female	105 (62.9 %)	0.51 ± 0.29	0.45−0.57	0.11−1.66	
**Age group**					0.426
18−30 years	92 (55.4 %)	0.48 ± 0.30	0.42−0.55	0.11−1.66	
31−40 years	30 (18.1 %)	0.53 ± 0.23	0.44−0.62	0.13−0.96	
41−50 years	27 (16.3 %)	0.57 ± 0.25	0.47−0.67	0.16−1.14	
>51 years	17(10.2 %)	0.45 ± 0.21	0.34−0.56	0.11−0.84	
**Region**					0.235
Beirut	74 (44.3 %)	0.53 ± 0.30	0.46−0.60	0.11−1.66	
Mount Lebanon	24 (14.4 %)	0.54 ± 0.25	0.43−0.64	0.15−1.14	
South	22 (13.2 %)	0.43 ± 0.27	0.31−0.55	0.13−1.34	
North	29 (17.4 %)	0.42 ± 0.17	0.35−0.49	0.12−0.87	
Bekaa	18 (10.8 %)	0.56 ± 0.29	0.42−0.71	0.18−1.15	

* CI - Confidence Interval; EFSA - European Food Safety Authority; SD - Standard Deviation.

**Fig. 1:**
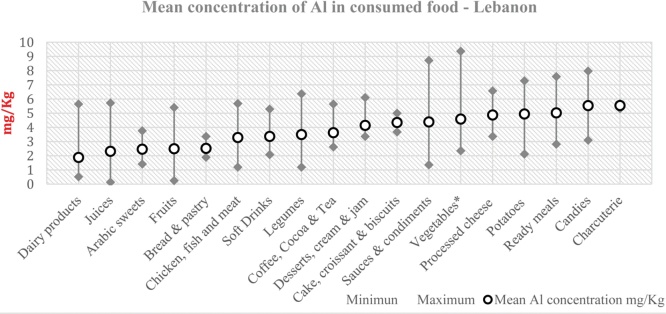
Distribution of Al level by mean with minimum and maximum range in analyzed food items.

97 food items were analyzed. 8 items were discarded for turbidity reasons. Analysis of samples was done in triplicate. The mean concentration of Al in food (mg/kg) is presented in Fig. 1, with a total mean of 3.56 ± 2.08 mg/kg ranging from (0.14 to 9.37). It was ghettoized as follows: between 5 and 6 (ready meals, candies, charcuterie); 4 and 5 (desserts, cream, jam, cake, croissant, biscuits, sauces & condiments, vegetables, processed cheese and potatoes); 3–4 (chicken, fish, meat, soft drinks, legumes, coffee, cocoa and tea); 2–3 (juices, Arabic sweets, bread, pastry, chicken, fish, meat, soft drinks, legumes, coffee, cocoa and tea) and around 1–2 (dairy products). The mean concentrations of Al in consumed foods (mg/kg) were presented by its climax, and we found that the top ranked mean concentration was found among Vegetables (9.37), followed by Sauce and Condiments (8.72), Candies (7.97), Ready Meals (7.58), Potatoes (7.28) and Coffee, Cocoa and Tea (6.36). The highest food ingestion rates (g/day) as shown in Table 3, were as followed for Soft Drinks (102.70), Fresh Tomatoes (75.57), Tea (71.43), Ice Cream (74.07), Turkish Coffee (60.41) and Fresh Lettuce (59.75). Studied items came into contact with Al mainly through cookware (9 items), packaging (63 items) and foil wrapping (5 items). The other 20 selected items might have had contact with Al naturally, from food additives and/or by preparation process therefore they were listed under unspecified.

**Table 3:** Distribution of analyzed food groups by Consumption Rate (CR), Portion Size (PS) and Ingestion Rate (IR) and their contact with Al.

Food groups	Al C**	CR*	PS*	IR*
**1-Arabic sweets (4)**				
Kanafeh (1)	CW	0.3031	170	51.53
Maamoul with pistachios (1)	CW	0.5891	50	29.46
Maamoul with dates (1)	CW	0.5891	50	29.46
Maamoul with walnuts (1)	CW	0.5891	50	29.46
**2- Bread and pastry (5)**				
Arabic bread (pita) (1)	UNS	1.2966	30	38.90
Baguette (1)	UNS	0.2625	100	26.25
Pizza (1)	UNS	0.0660	50	3.30
Fatayer (1)	UNS	0.0356	50	1.78
Mankushe (Zaatar) (1)	UNS	0.1983	100	19.83
**3-Cake, croissant and biscuits (3)**				
Chocolate muffin (1)	P	0.1796	50	8.98
Croissant (1)	P	0.0855	57	4.87
Chocolate wafer (1)	P	0.1606	35	5.62
**4- Candies (2)**				
Candies (2)	P	0.2110	20	4.22
**5- Charcuterie (4)**				
Mortadella beef (1)	P	0.0322	57	1.84
Mortadella chicken (1)	P	0.0592	57	3.37
Mortadella turkey (1)	P	0.0322	57	1.84
Hot dogs (1)	P	0.0042	35	0.15
**6- Chicken, fish and red meat (6)**				
Rotisserie chicken (1)	FW	0.1204	105	12.64
Grilled chicken (1)	FW	0.1038	105	10.90
Fried escalope (1)	UNS	0.2907	105	30.52
Grilled fish (1)	FW	0.0373	100	3.73
Sardines (1)	P	0.0140	57	0.80
Barbecue (1)	CW	0.0753	120	9.04
**7- Coffee, Cocoa & Tea (7)**				
Capsuled coffee (1)	P	0.2251	80	18.01
Turkish coffee (1)	CW	0.7551	80	60.41
In sachet instant coffee (2)	P	0.4916	80	39.33
Hot chocolate (1)	P	0.0684	100	6.84
Cocoa powder (1)	P	0.0684	100	6.84
Tea (1)	UNS	0.4762	150	71.43
**8- Dairy products (11)**				
Full fat milk (4)	P	0.1713	250	42.83
Powdered milk (1)	P	0.1456	45	6.55
Labneh (1)	P	0.5747	50	28.74
Yogurt drink (4)	P	0.1062	225	23.90
Unsalted butter (1)	P	0.307	15	4.605
**9- Desserts, cream and jam (5)**				
Whipped cream (1)	P	0.0322	15	0.48
Sweetened condensed milk (1)	P	0.0302	15	0.45
Cooking cream (1)	P	0.0376	15	0.56
Ice cream (1)	P	0.7407	100	74.07
Canned strawberry jam (1)	P	0.0187	30	0.56
**10- Fruits (3)**				
Fresh apple (1)	UNS	0.4804	100	48.04
Fresh plum (1)	UNS	0.3018	100	30.18
Canned fruit salad (1)	P	0.0038	60	0.23
**11- Juices (11)**				
Packed juice (8)	P	0.2059	200	41.18
Canned juice (2)	P	0.2059	200	41.18
Powdered juice (1)	P	0.0807	45	3.63
**12- Legumes (4)**				
Foul (fava beans) (1)	CW	0.3211	100	32.11
Hommos (chick peas) (1)	CW	0.3211	100	32.11
Canned lentil (1)	P	0.2706	100	27.06
Canned corn (1)	P	0.0968	100	9.68
**13- Potatoes (6)**				
Sachet of chips (3)	P	0.0459	34	1.56
Fried potatoes (1)	CW	0.0937	80	7.50
Grilled potatoes (1)	FW	0.0493	80	3.94
Baked potatoes (1)	FW	0.0493	100	4.93
**14- Processed cheese (3)**				
Packed melted cheese (3)	P	0.2283	60	13.70
**15- Ready meals (5)**				
Shawarma (1)	UNS	0.3543	90	31.89
Falafel (1)	UNS	0.3543	80	28.34
Basterma (1)	UNS	0.0281	100	2.81
Makanek (Beef sausages) (1)	UNS	0.0281	100	2.81
Cooked rice (1)	UNS	0.0629	100	6.29
**16- Sauces & condiments (7)**				
Ketchup (1)	P	0.2402	15	3.60
Cucumber pickles (1)	UNS	0.1689	30	5.07
Onion sauce (1)	UNS	0.1205	15	1.81
Hot chili sauce (1)	UNS	0.1205	15	1.81
Tomato paste (1)	P	0.2080	15	3.12
Cinnamon spices (1)	P	0.1328	15	1.99
Chicken broth cubes (1)	P	0.1328	5	0.66
**17- Soft Drinks (6)**				
Orange, cola, lemon (6)	P	0.4279	240	102.70
**18- Vegetables excluding potatoes (5)**				
Fresh carrots (1)	UNS	0.2996	100	29.96
Fresh lettuce (1)	UNS	0.5975	100	59.75
Fresh tomatoes (1)	UNS	0.7557	100	75.57
Canned peas (1)	P	0.0362	100	3.62
Canned mushroom (1)	P	0.0947	100	9.47
**Total (97)**				

*CR – Consumption Rate /day.

PS – Portion Size (g).

IR – Ingestion Rate (g/day).

**Al C – Al contact: CW – Cooking ware; P – Packaging; FW – Foil wrapping; UNS – Unspecified.

For the estimation of Al exposure from selected food, and compared with data reported by international agencies and based on a body weight of 60 kg, DWI of Al in the study population expressed in mg/kg bw/day was estimated to 0.50 (50th DWI) and 1.01 (95th DWI); mg/kg body weight per week. DWI was statistically significant compared with the international standards of the EFSA 1 mg/kg, p < 0.001 – Table 2. While we found an escalation of DWI means from 0.48 to 0.57 respectively between the age groups of 18−30 and 41–50 years. Participants from South and North regions had lower DWI means (0.43 and 0.42). The highest means were found in Bekaa (0.56). Females showed a slightly higher mean of DWI compared to males (0.51 vs 0.49), but there was no statistical difference among gender, age groups and region for DWI (Table 2).

## 4 Discussion

Selected food items were presented in different forms: fresh from market; processed; canned; cooked food. Therefore, their contamination with Al came from different sources; mainly from soil, being processed and cooked, and/or being packed and canned. They may have been contaminated from external sources such as food additives (Al derivatives as defined by the food industry) or by cooking and packaging, knowing that Al interacts with acidic compounds. 20 food groups out of 26 (76.9 %) and 84 food items out of 97 (86.5 %) had a mean concentration of Al below 5 mg/kg (Fig. 1).

In our study, Al concentration in food varied from 0.14 to 9.37 mg/kg of fresh weight. The major factors of food contaminant by Al were not specified, but soil and irrigation water appear to have been the primary cause of contamination. The content of Al in food showed large dissimilarities between countries. This was due to the variation in the study design, selected food, food processing (diverse quantities used for Al containing food additives), cooking, storage (contact with aluminum utensils, containers, Al foil and others) and the method used in laboratory analysis (preparation and analysis procedures for example using fresh or dry weight, microwave or acid digestion, Inductive Coupled Plasma spectrometry or Atomic Absorption Spectrometry technique used) that might affect the quantification of Al (basically inherent in food and/or accumulated via contamination). Therefore, comparative study might have several limitations. Nevertheless, the detection of Al in the selected food groups have shown almost similar levels to other studies [40,41].

To evaluate the food exposure of dietary Al, several countries have used an individual food approach in their Total Diet Survey (TDS). In our study, following the previously mentioned approach; the selection of food was not holistic and only food items known to have high levels of Al (based on literature review and specifically from the results of the 2nd French Total Diet Study) were included in the quantification. Therefore, the Daily Dietary Intake (DDI) to Al by the Lebanese population was estimated to 71.4 μg/Kg bw/day (estimating a typical body weight of 60 Kg per person), similar to findings from Italy and the UK (60 vs 71 μg/Kg bw/day) [42,43]. Meanwhile, we found that for other countries which had followed the same methodology of TDS, DDI means were classified as follows: Australia 36.0; France 40.3; Spain 170; and Canada 105–173 μg/Kg bw/day (with difference in bw calculation) [11]. These results could suggest Lebanon is on the ‘safer side’, since the exposure to Al in the analyzed consumed food by the Lebanese population falls below the thresholds of international agencies. While this is encouraging, the fact of high Al DDE in Lebanon’s vegetables, sweets and ready meals indicate a need for serious consideration regarding food safety monitoring, and control for soil-based Al contamination. In addition, attention to integrate new approach for risk assessment might be well-thought-out [44,45].

Many challenges had to be overcome while working with our food matrices. Since this is the first study to evaluate Al contamination in food in Lebanon, we anticipated extensive Al contamination. Still, we took all necessary precautions and followed much guidance and recommendations to quantify and analyze the metal present. With the improvement of technology, it was difficult to move from ordinary TDS studies to a selective metal study using a simplified E-FFQ (with the additions of both portion sizes and tailored photos), but this enabled us to have low study cost and low timing for data collection. The methodology we followed using Curve® helped us to collect and convert our data with minimal biases in a relatively short time. As justification, it is known that the highest consumption of soft drinks worldwide is found in the Middle East and therefore proven to be compatible with our study’s outcome [46].

This study had some limitations as purchased food items were randomly selected from Beirut market, which limits the inclusion of all Lebanese areas surveyed within the study. The number of purchased food items and their analysis has been restricted to essential food due to the lack of financial resources, and the selection may not be exhaustive to test the consumption of all food containing Al. The adopted E-FFQ followed Lebanon FBDG, but still cannot be completely excluded of memory bias, as well as the over-reporting and under-reporting of food consumption. The acid digestion of selected food samples in our study started from a fresh weight of 5 g, while other studies used end weight, which could be set as dry weight and may affect the comparison of our results to others.

## 5 Conclusion

The investigated food in our study, retrieved from the Lebanese market, presented the highest concentration of Al in Vegetables, followed by Sauces and Condiments, Candies and then Ready Meals. The highest food exposure to Al in the Lebanese population comes from lettuce, soft drinks, ice cream, tea and packed juices. Al intake compared with the established PTWI by international agencies showed an estimated mean of 0.50 mg/kg bw, which does not exceed the international limit. In Lebanon, efforts should focus to set recommendations to manage and control the presence of Al in food. Safety measures should be put in place to limit the usage of Al lining in packaged foods (especially sauces and condiments, ice cream and juice), to limit the use of Al foil and utensils in cooking, and the consumption of canned food, as Al leaches from the containers into their contents. The contamination of vegetables by Al might be due to polluted soil caused by acid rains, or by water used for irrigation, and thus further investigation should be considered.

## Author statement

The author confirm that this work is original and has not been published elsewhere, nor is it currently under consideration for publication elsewhere.

This paper is the first national study to explore Aluminum (Al) presence in food and its exposure by the Lebanese population. Upon observation many traditional food are cooked in Al utensils and are in contact with the metal. We have followed the results of the French Total Diet study and used a unique methodology to investigate Al containing food consumption by using an e-FFQ via Curve platform. We have collected food from market in different forms, studied its metal level and estimated the exposure of the Lebanese population to Al comparing it to other countries. The estimated Provisional Tolerable Weekly Intake does not exceed the limit established by international agencies. Recommendations were set to manage and control the presence of Al in food. We shed the light on the contamination of vegetables by the metal that might be due to polluted soil by acid rains or by the water used for irrigation, therefore further investigation should be considered.

## Funding and acknowledgment

This work was supported by the 10.13039/501100004113Lebanese University and Nantes University. We acknowledge Dr Joseph Matta from IRI Laboratory for assisting us with the food analysis process, and Syscom Technologies for helping in creation of the E-FFQ and creating the Curve® platform.

## CRediT authorship contribution statement

**Sarine EL Daouk:** Conceptualization, Methodology, Software, Data curation, Writing - original draft. **Alain Pineau:** Data curation, Writing - original draft. **Mariam Taha:** Writing - review & editing. **Raed Ezzeddine:** Writing - review & editing. **Akram Hijazi:** Investigation, Supervision. **Mohamad Al Iskandarani:** Visualization, Investigation, Validation, Supervision, Writing - original draft.

## Declaration of Competing Interest

The authors declare that they have no known competing financial interests or personal relationships that could have appeared to influence the work reported in this paper.
